# Supplementary material for: Vortex Fluidic Mediated Oxidative Sulfitolysis of Oxytocin
Source: Molecules. 2022 Feb 7;27(3):1109. doi: 10.3390/molecules27031109 (PMC8840205; doi:10.3390/molecules27031109)
Supplement: Supplementary file 1 [file molecules-27-01109-s001.zip › molecules-1582484-supplementary.pdf]

molecules

Supplementary Material

Vortex Fluidic Mediated Oxidative Sulfitolysis of Oxytocin

Emily Crawley [1], Scott Pye [1], Briony Forbes [2] and Colin Raston [1,*]

[1] Flinders Institute for Nanoscale Science and Technology, College of Science and Engineering, Flinders University, Bedford Park, South Australia 5042, Australia;
[2] College of Medicine and Public Health, Flinders University, Bedford Park, South Australia 5042, Australia;
* Correspondence: colin.raston@flinders.edu.au

The vortex fluidic device (VFD) shown in Figure S1 is modular in nature, thus this device can have many configurations. Throughout this report the following specifications were used. The tube used was borosilicate glass, 19 cm long with a 20 mm outer diameter (OD). Rotating tube was always operated at a 45° tilt angle (θ).

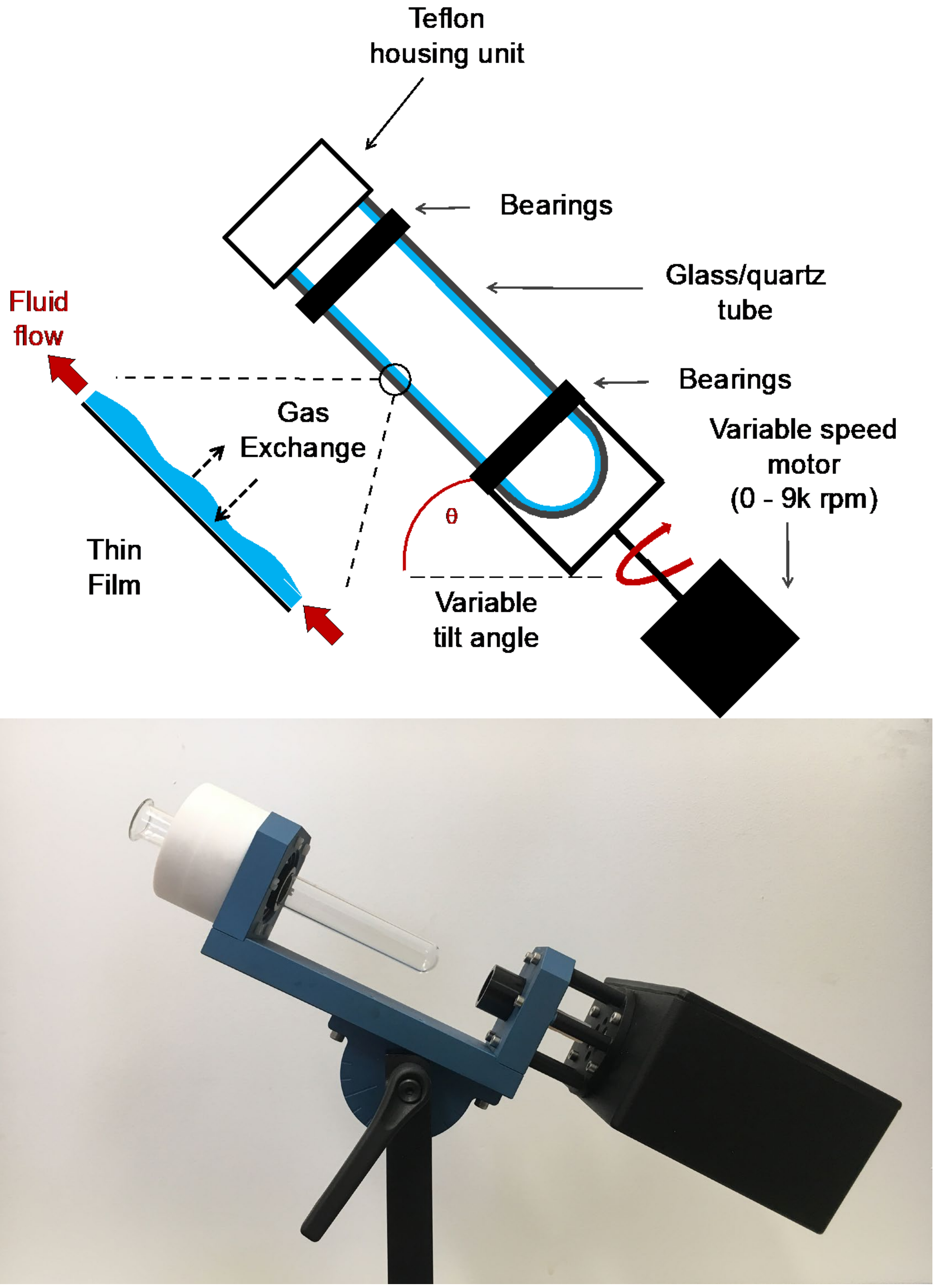

Figure S1. Schematic representation and photograph of the vortex fluidic device (VFD).

Oxytocin

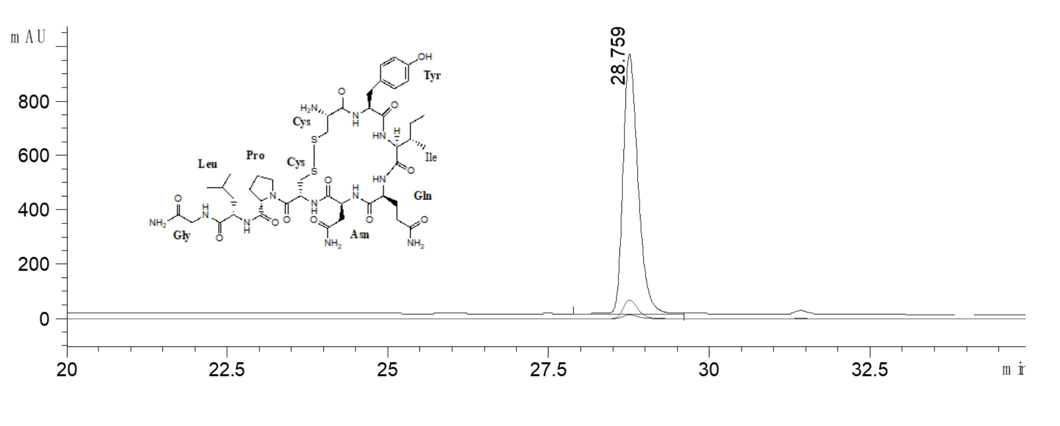

Oxytocin; t_R = 28.76 min

(**a**)

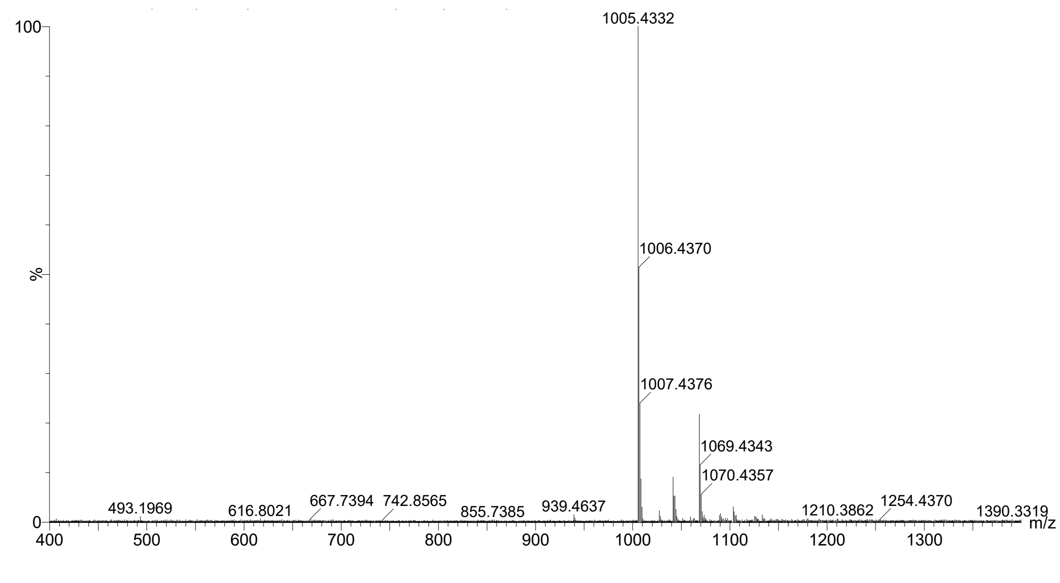

Oxytocin $[M-H]^-$ = 1005.48 m/z

(**b**)

Figure S2. (a) HPLC chromatogram and (b) mass spectra of the eluent peak at 28.76, matching native oxytocin.

Sulfitolysed oxytocin

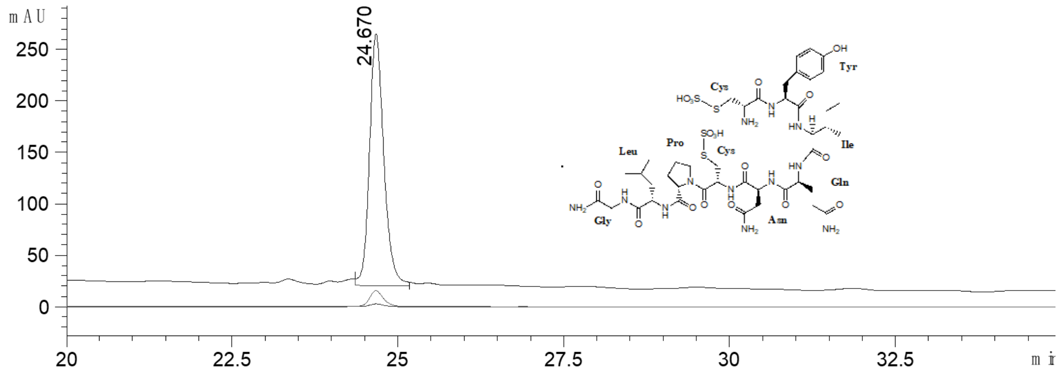

Sulfitolysed oxytocin; t_R = 24.67 min

(**a**)

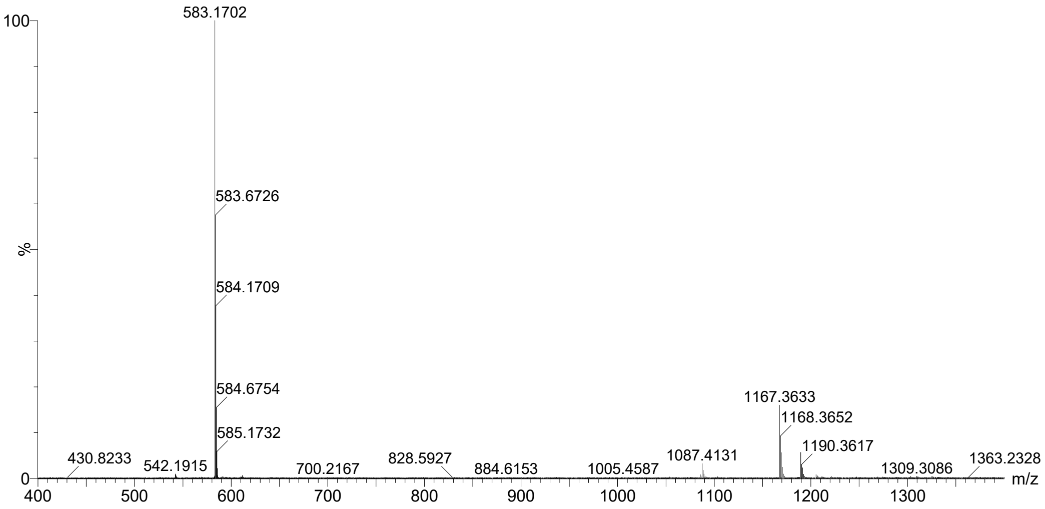

Sulfitolysed oxytocin $[M-H]^-$ = 1167.4 m/z

(**b**)

Figure S3. (a) HPLC chromatogram and (b) mass spectra of the eluent peak at 24.67 mins, matching sulfitolysed oxytocin.
